# Characterization of Auditory and Binaural Spatial Hearing in a Fragile X Syndrome Mouse Model

**DOI:** 10.1523/ENEURO.0300-19.2019

**Published:** 2020-01-30

**Authors:** Elizabeth A. McCullagh, Shani Poleg, Nathaniel T. Greene, Molly M. Huntsman, Daniel J. Tollin, Achim Klug

**Affiliations:** 1 Department of Physiology and Biophysics, University of Colorado Anschutz, Aurora, Colorado 80045; 2 Department of Otolaryngology, University of Colorado Anschutz, Aurora, Colorado 80045; 3 Departments of Pediatrics and Pharmaceutical Sciences, University of Colorado Anschutz, Aurora, Colorado 80045; 4 Department of Integrative Biology, Oklahoma State University, Stillwater, Oklahoma 74074

**Keywords:** acoustic startle response, binaural hearing, fragile X syndrome, prepulse inhibition, spatial acuity

## Abstract

The auditory brainstem compares sound-evoked excitation and inhibition from both ears to compute sound source location and determine spatial acuity. Although alterations to the anatomy and physiology of the auditory brainstem have been demonstrated in fragile X syndrome (FXS), it is not known whether these changes cause spatial acuity deficits in FXS. To test the hypothesis that FXS-related alterations to brainstem circuits impair spatial hearing abilities, a reflexive prepulse inhibition (PPI) task, with variations in sound (gap, location, masking) as the prepulse stimulus, was used on *Fmr1* knock-out mice and B6 controls. Specifically, *Fmr1* mice show decreased PPI compared with wild-type mice during gap detection, changes in sound source location, and spatial release from masking with no alteration to their overall startle thresholds compared with wild-type mice. Last, *Fmr1* mice have increased latency to respond in these tasks, suggesting additional impairments in the pathway responsible for reacting to a startling sound. This study further supports data in humans with FXS that show similar deficits in PPI.

## Significance Statement

This is the first study to characterize auditory spatial acuity in a mouse model of FXS. We saw minor differences in *Fmr1* mice compared with B6 mice in several measures of auditory acuity as measured by inhibition of the startle response. *Fmr1* mice had increased latency to startle for almost all conditions compared with B6 mice, suggesting altered timing to acoustic cues. These experiments further show that, consistent with patient report and anatomic/physiologic data, the auditory system is altered in a mouse model of FXS, though with some potential compensation leading to a subtle behavioral impact.

## Introduction

Fragile X syndrome (FXS) is the leading monogenetic cause of autism (Penagarikano et al., 2007). FXS is caused by a mutation in the gene *Fmr1* that encodes fragile X mental retardation protein (FMRP). One of the hallmark symptoms of FXS, among many other cognitive symptoms, is auditory hypersensitivity (for review, see Rotschafer and Razak, 2014). An imbalance of neural excitation/inhibition (E/I) is thought to underlie many pathologies in FXS (Contractor et al., 2015), including those leading to auditory symptomology (Keine et al., 2016).

E/I imbalances in FXS extend to the auditory brainstem circuits responsible for sound localization, as well as auditory cortical areas (Rotschafer et al., 2015; Garcia-Pino et al., 2017; McCullagh et al., 2017; Rotschafer and Cramer, 2017). FMRP is highly expressed in the auditory brainstem (Wang et al., 2014; Ruby et al., 2015; Zorio et al., 2017), leading to changes in potassium channel distribution (Brown et al., 2010; Strumbos et al., 2010) that underlie changes in synaptic function *in vitro* (Wang et al., 2015; Garcia-Pino et al., 2017; Curry et al., 2018; El-Hassar et al., 2019; Lu, 2019). In addition, studies have shown alterations to the auditory brainstem response (ABR), an *in vivo* measure of auditory brainstem activity, in *Fmr1* mice that are potentially caused by these underlying changes to E/I balance and physiologic activity (Rotschafer et al., 2015; El-Hassar et al., 2019).

Binaural hearing and spatial acuity not only used for sound localization per se but are also essential for communication in busy acoustic environments where several sound sources are active at the same time, which in the literature often labeled “cocktail party situations” (Cherry, 1953). In such situations, the sound localization pathway in the auditory brainstem associates these various sounds with their respective spatial channel, thereby providing the foundation for our ability to follow a particular sound of interest when competing sounds are present. This separation is dependent on an intricate E/I balance that starts in the auditory brainstem (for review, see Grothe et al., 2010; Bronkhorst, 2015). Basic encoding of sound location information is also relayed through the precise balance of E/I and is encoded as interaural level differences (ILDs) and interaural timing differences (ITDs; Pollak et al., 2002; Grothe et al., 2010). ITDs and ILDs are dependent on timing and level information from the two ears that becomes excitation or inhibition within the auditory brainstem (Goldberg and Brown, 1969; Caird and Klinke, 1983; Moore and Caspary, 1983; Thompson and Schofield, 2000). Therefore, impairments in the E/I balance of the auditory brainstem that occur in FXS are expected to lead to impaired ability to function in complex noisy acoustic environments and ITD and ILD encoding. As a signal is displaced further in space from a distracting background noise, it becomes easier to discriminate from the noise; this effect is termed spatial release from masking (SRM; for review, see Feng and Ratnam, 2000). Despite the substantial alterations to the auditory brainstem in FXS, it has never been clearly shown, beyond patient report and surveys, that mice or humans with a mutation in *Fmr1* have impairments in their ability to localize sound in normal listening or complex acoustic environments (Baranek et al., 2002, 2008; Rogers et al., 2003).

This study tests the hypothesis that mice with a mutation in the *Fmr1* gene have a functional deficit in binaural hearing despite a normal range of auditory hearing ability. Binaural hearing ability was assessed using a reflexive prepulse inhibition (PPI) paradigm, where a change in the sound source location served as the prepulse, a method described previously for mice (Allen and Ison, 2010) and guinea pigs (Greene et al., 2018). The acoustic startle response (ASR) is a reflexive whole-body response elicited by a very brief, but loud impulse noise. PPI consists of modification of the acoustic startle response by pairing the startle-eliciting stimulus with a preceding stimulus, the prepulse, that inhibits the startle response by providing a cue to the impending startle (Young and Fechter, 1983). PPI is a useful tool for measuring deficits in cognitive disorders such as autism, FXS, or schizophrenia since it is reflexive and independent of cognitive ability (Young and Fechter, 1983). It has been shown previously, with conflicting results, that mice and humans with *Fmr1* mutations have impaired PPI (Chen and Toth, 2001; Nielsen et al., 2002; Frankland et al., 2004; Hessl et al., 2009; Thomas et al., 2012; Veeraragavan et al., 2012), suggesting altered sensorimotor gating. We use a similar PPI paradigm, but with cues to specifically target spatial hearing ability (gap in sound, speaker swaps, and spatial release from masking), to test the hypothesis that behavioral impairments result from altered signaling in the brainstem sound localization circuits. We show subtle changes in *Fmr1* mice responses to spatial auditory stimuli, quantified as reduced PPI, reduced detection of prepulses compared with wild-type animals, and increased response latencies to startling sounds.

## Materials and Methods

All experiments complied with all applicable laws, National Institutes of Health guidelines, and were approved by the University of Colorado Anschutz Institutional Animal Care and Use Committee.

### Subjects

All experiments were conducted in either C57BL/6J background (wild type) or hemizygous male and homozygous *Fmr1* knock-out strain maintained on the background [B6.129P2-*Fmr1^tm1Cgr^*/J, *Fmr1*, stock #003025 (Dutch-Belgian Fragile X Consortium, 1994); C57BL/6J stock #000664, The Jackson Laboratory]. A total of 46 mice were used, and the exact number of animals used per experiment are listed in the figure legend and corresponding Results sections. The results are based on experiments conducted in both male (*N* = 30) and female (*N* = 16) wild-type mice and *Fmr1* knock-out mice. No significant differences were observed between sexes, and data for males and females were combined. Animals were genotyped regularly using Transnetyx. Mice used in these experiments were all adult animals and varied in age between 55 and 167 d. Age was not significantly different between the two groups (*p* = 0.96; *Fmr1* mice, 83.1 ± 4.3 d old; wild-type mice, 83.4 ± 4.7 d old). Each animal was weighed after the completion of data collection. Animal weights varied between 13 and 37 g, and generally increased with age. On average, *Fmr1* mice tended to weigh less (23.5 ± 0.75 g) than wild-type animals (25.8 ± 0.43 g; *p* = 0.01). Pinna morphology was measured using methods described in the study by Anbuhl et al. (2017) by measuring the height and width of each ear and estimating the effective diameter (square root of the height × width). There was no significant difference in pinna morphology between B6 and *Fmr1* animals (*p* = 0.9026; diameter: B6, 8.79 ± 0.36; *Fmr1*, 8.84 ± 0.21).

### ABR audiogram

ABR recordings were conducted using methods similar to those described previously (Ferber et al., 2016; Benichoux et al., 2018). Briefly, a devoted cohort of mice (seven mice of each genotype; B6 of both sexes, 132 ± 2.8 d old; *Fmr1* of both sexes, 117.9 ± 4.3 d old) were anesthetized (60 mg/kg ketamine + 10 mg/kg xylazine for initial anesthesia and 25 mg/kg ketamine + 12 mg/kg xylazine) and placed on a heating pad in a small sound-attenuating chamber (ETS-Lindgren). Stimuli presentation and generation as well as recording of evoked potentials were conducted through custom MATLAB (MathWorks) software interfacing with an RME Fireface UCX Sound Card (operating at a sampling rate of 44.1 kHz). Stimuli were presented through calibrated (Beutelmann et al., 2015) Etymotic ER10B+ ear coupling tubes and were presented by ER2 earphones using standard sound delivery tubes. Evoked potentials were made with platinum subdermal needle electrodes (F-E2-12 electrodes, Grass Technologies) simultaneously at the apex, and behind each pinna (active), referenced to the nape of the neck, with a hindleg ground. Signals were amplified and digitized by a Tucker Davis Technologies (TDT) low-impedance headstage (RA4LI) and preamplifier (RA4PA), further amplified digitally (10,000×), and output as an analog signal by a multi-input/output processor (RZ5). ABRs were recorded in response to 1000 repetitions of short (5 ms) tone-burst (4, 8, and 16 kHz) stimuli, gated on and off with 1 ms linear ramps, and presented at a rate of ∼14/s. Frequencies of the ABR stimuli spanned the effective bandwidth of the auditory stimuli used for behavioral testing (see next section). Stimuli included interleaved presentations of left and right monaural, as well as binaural stimulus presentations. Responses were initially recorded for 90 dB SPL stimulus presentations, then for progressively lower levels (in 10 dB SPL steps) until the threshold was found. Threshold was determined as the average between when a wave form was present and the next dB SPL step in which a discernable ABR signal appeared. Responses were analyzed by assessing for the lowest level eliciting a detectable evoked potential in any of the three recorded channels.

### Apparatus

Experimental conditions and apparatus have been previously described in the study by Greene et al. (2018) but are also briefly described here. All experiments were conducted in a double-walled sound attenuating chamber (IAC Acoustics) lined with acoustical foam to reduce echoes. The animal was snugly placed, to ensure the animal was forward facing, in a custom-built acoustically transparent steel-wired cage attached to a polyvinyl chloride post anchored to a flexible polycarbonate platform with an accelerometer (catalog #ADXL335, Analog Devices) to capture startle responses. All animals were tested in the dark using a closed-circuit infrared camera to monitor movement and proper orientation of the animal. When the animal was placed snugly into the chamber with the steel lightly compressed around its body, the animal maintained its forward-facing position and was unable to turn around. The cage with the animal was then always oriented toward the center loudspeaker. A diagram with the experimental apparatus is shown in the study by Greene et al. (2018). The chamber consisted of an array of 25 loudspeakers (model MDT-20, Morel) placed horizontally in a 1-m-radius semicircular boom at 7.5° intervals from −90° (right) to +90° (left) in front of the animal from which prepulse stimuli were presented. Startle stimuli were presented from a Faital Pro HF102 compression driver placed ∼35 cm (from the base of the platform) directly above the cage and amplified using an Alesis RA150 amplifier.

Stimuli were generated and responses recorded from three TDT RP 2.1 Real-Time processors using custom-written MATLAB (MathWorks) software. The startle stimuli were 20 ms broadband noise bursts generated by one of the RP2.1 processors and presented at 110 dB SPL (unless otherwise stated, as in the startle threshold experiments). Carrier stimuli (CSs) were broadband-noise generated by a second RP2.1 and presented continuously (unless otherwise noted) during testing. In the speaker swap experiment, the broadband noise was high-pass filtered (4 kHz cutoff) with a 100th order FIR (finite impulse response) filter. Because the Morel MDT-20 loudspeakers begin to roll off at 20 kHz the effective bandwidth of the noise stimuli was ∼4–20 kHz. The CS was presented from one speaker at a time and had the ability to be switched by two sets of TDT PM2Relay power multiplexers controlled by the RP2.1s. Attenuation of the signals and startle stimuli was achieved using TDT PA5 programmable attenuators. Vertical movement of the polycarbonate plate on which the accelerometer was mounted was detected as the voltage output, sampled at 1 kHz by one of the RP2.1 processors. Startle response amplitude was calculated as the root mean square (rms) of the accelerometer output in the first 100 ms after the delivery of the startle stimuli, and startle latency was calculated as the delay between the startle stimulus onset and the time at which the accelerometer output exceeded three SDs of the 100 ms immediately preceding the startle stimulus presentation.

### Experimental conditions

Four types of experiments were conducted on most of the animals: startle threshold, gap detection, speaker swap, and spatial release from masking (similar to experiments performed in studies by Allen and Ison, 2010; Greene et al., 2018). The first repetition within a condition was excluded from analysis, and all conditions were presented at least four times per experiment, with most experiments containing six or more trials per condition. Most of the animals were tested once in each experiment; however, some mice were not subjected to acoustic startle threshold testing to reduce overall data collection time. The order of each of the four types of experiments was pseudorandomized for each animal, and each animal was only tested once per experiment (e.g., startle, gap). Total time of testing was ∼3 h per animal, and all conducted on the same day. The intertrial interval (i.e., the time between trials) was uniformly distributed between 15 and 25 s in 1 s increments to prevent the animal from acclimating to the time of startle. For all experiments excluding the startle threshold, the startle stimulus was presented at 110 dB SPL. The order of prepulse conditions was pseudorandomly presented for each experiment.

### Experiment 1: startle threshold

Startle threshold was assessed by varying the intensity (for most animals, between 60 and 120 dB SPL in 10 dB steps) of the startle-eliciting stimulus, presented with the overhead startle speaker and recording their ASR through the cage-mounted accelerometer. Startle responses were assessed in the presence of a 70 dB SPL background noise played continuously from the speaker directly in front of the animal (0°). Presentation of these conditions was limited to three to five repetitions to ensure that the animal had a robust startle response while minimizing the duration of testing.

### Experiment 2: gap detection

The ability of animals to detect a short quiet period in a continuously noisy background was similarly assessed by presenting a broadband noise from the speaker directly in front of the animal (0°). A 20 ms gap in the noise (the prepulse) was introduced before the startle-eliciting stimulus with interstimulus intervals (ISIs; time between the stimulus (gap) and startle-eliciting stimulus; see Fig. 3*A*) of 1, 2, 5, 10, 20, 40, 80, 160, and 240 ms from the onset of the gap. A subset of animals was only tested with 10, 20, 40, 80, 160, and 240 ms ISIs. Responses were assessed for 10 repetitions of each ISI, presented pseudorandomly in a blockwise fashion. Two control condition trials, consisting of the continuous broadband noise with no gap preceding the startle-eliciting stimulus, were included in each repetition block.

### Experiment 3a: fixed 90° angle speaker swap with variable interstimulus interval

The optimal ISI for speaker swap detection was assessed by swapping the source speaker (the prepulse) of a continuous broadband noise (70 dB SPL) 90° symmetrically across the midline (see Fig. 4*A*). The background noise was initially played from the speaker −45° (right) with respect to the animal and swapped with the speaker +45° (left) of the animal at some ISI before the startle-eliciting stimulus. Startle responses were assessed for five repetitions of 1, 2, 5, 10, 20, 30, 40, 80, 100, 150, and 300 ms ISIs, presented randomized in a blockwise fashion. Two control conditions, where no speaker swap occurred (i.e. the noise was continuously played from the initial speaker at −45°), were included in each repetition block. The ISIs for gap and speaker swap detection may be different, as well as the number of repetitions needed, and therefore different ISIs and numbers of repetitions were presented for experiments 2 and 3a.

### Experiment 3b: variable angle speaker swap with 20 ms fixed ISI

Minimum audible angle was similarly assessed using a speaker swap paradigm. The animal orientation was maintained at 0° (center), as described above, to test responses to sounds swapped across the midline and to assess minimum audible angle detection ability. The prepulse was a change in the source of a high-pass noise (cutoff, <4 kHz) between two matched speakers separated by 7.5°, 15°, 30°, 45°, and 90° symmetrically (except for 7.5°) across the midline, in both directions (left to right and right to left). The ISI was set at 20 ms between presentation of the prepulse (speaker swap) and the startle-eliciting stimulus. The ISI was set in this experiment to reduce the length of the overall experimentation time and based on the results from experiments 3a and 2 showing that an ISI of 20 ms is optimal for eliciting PPI. Startle responses were assessed for eight presentations of each condition (*N* = 10 since swap angle and direction covary), and one control condition (the high-pass noise presented from the starting speaker, but no swap to the matched speaker) for each starting speaker (*N* = 10), was presented randomized within repetition blocks.

### Experiment 4a: detection threshold for spatial release from masking

The detection threshold of the signal used in an SRM (i.e., the ability of mice to detect a signal in a continuous 70 dB SPL broadband masking noise presented from the center speaker; 0°) was assessed by varying the intensity of the “signal” speaker presented adjacent to the center speaker (7.5°, SRM threshold; see Fig. 6*A*). The ISI was set at 20 ms from the onset of the prepulse to the startle-eliciting stimulus. The signal was a 100 ms duration multitone complex with a 4 kHz fundamental frequency and overtones at octave spacing up to 32 kHz (4 octaves). The intensity of the signal was varied by decreasing the signal level by 9, 12, 15, 18, 21, 24, and 27 dB attenuation relative to the full scale (∼83.5 dB SPL), with a TDT PA5 programmable attenuator. Two control conditions (in which the masking noise was presented continuously with no signal prepulse presented) were included in each of five randomized trial blocks.

### Experiment 4b: speaker swap spatial release from masking

SRM was assessed by varying the location of the signal speaker at two levels determined based on preliminary results from the detection threshold task. In this task, the same signal as in experiment 4a was presented at 15 or 24 dB attenuation (from ∼83.5 dB SPL; see Fig. 7*A*), from speakers at 7.5°, 15°, 30°, 45°, and 90° (to the left or right) relative to center (0°) at a constant ISI of 20 ms. Two control conditions (in which the masking noise was presented continuously with no signal prepulse presented) were once again included in each randomized repetition block (of five).

### Data analysis

The ASR was assessed as the rms output of the accelerometer, amplified by 25 dB in the 100 ms following the startle-eliciting stimulus presentation. The units of the ASR are reported as arbitrary voltage units that are proportional to meters per second since the output of the accelerometer was not explicitly calibrated (though it was held constant throughout data collection). The mean ASR was calculated for each animal, with the first presentation of each condition excluded to exclude initial adaptation to the startle. Most responses were quantified as PPI, calculated as 1 minus the ratio of the mean prepulse ASR during each prepulse condition (ASR_p_) to the mean ASR during the corresponding control condition (ASR_c_), recorded for each session, as follows: PPI = 1 − [ASR_P_/ASR_c_]. A PPI of 0 corresponds to an ASR_p_ equal to ASR_c_, suggesting no detection of the prepulse, whereas both positive PPI, indicating a reduction in ASR, and negative PPI, indicating an increase in ASR, suggest that the prepulse was detected and modified the startle response of the animal. Figures were generated in R (R Core Team, 2013) using ggplot2 (Wickham, 2016). Data were analyzed using a mixed-effects model to account for repeat observations within one animal (lme4; Bates et al., 2015) with genotype and conditions (e.g., dB SPL, ISI, angle, ABR threshold) as fixed effects, and animal as a random effect. It was expected that there would be no differences between some conditions where the prepulse was not detectable. Therefore, a priori, independent of results from fixed effects (i.e., no difference in the main effect of genotype), it was determined that estimated marginal means (*emmeans*; Lenth, 2019) were going to be used to make pairwise comparisons between genotype and condition or replicate and condition. A Tukey method for multiple comparisons was implemented for these contrasts using *emmeans*. A zero-intercept model was used to compare genotypes to a PPI value of zero (to determine detection of the sound prepulse). The *t* tests used Satterthwaite’s method for comparing the multiple levels of ISI across genotype (Kuznetsova et al., 2017). Animal weight and age were compared between the two genotypes using a two-tailed *t* test, and data are presented as the mean ± SE. Tables 1-Tables 3 show mean, SE, median, interquartile range (IQR), and *p* values for each experiment. Bold values in Table 2 indicate conditions where PPI was >0. Asterisks are used to indicate statistical significance between the two genotypes, as follows: *p < 0.05, **p < 0.01, and ***p < 0.001. Figures were prepared for publication using Photoshop and Illustrator (Adobe).

**Table 1:** Summary statistics for ABR measurements

		*B6* mice			*Fmr1* mice			
Experiment		Mean ± SEM	Median	IQR Q1–Q3	Mean ± SEM	Median	IQR Q1–Q3	*P* Value
**ABR audiogram** **(dB SPL)**	4000	59.3 ± 3.7	55.0	55.0–65.0	61.7 ± 2.1	65.0	57.5–65.0	0.6278
	8000	39.3 ± 36.1	35.0	35.0–45.0	45.0 ± 3.7	45.0	37.5–52.5	0.2503
	16,000	37.9 ± 3.6	35.0	35.0–40.0	53.3 ± 3.1	55.0	47.5–55.0	0.0040

**Table 2:** Summary statistics for startle threshold and PPI experiments

		*B6* mice			*Fmr1* mice			
Experiment		Mean ± SEM	Median	IQR Q1–Q3	Mean ± SEM	Median	IQR Q1–Q3	*p* Value
**Threshold (dB SPL)**	60	329.0 ± 13.7	289.0	267–369	380.1 ± 36.8	312.5	272–412	0.2903
	70	387.0 ± 36.1	320.0	276–363	349.0 ± 22.3	306.0	273–360	0.0706
	80	391.0 ± 17.6	326.0	273–489	457.3 ± 37.8	320.5	266–539	0.8195
	90	633.8 ± 33.9	586.0	397–758	731.3 ± 69.2	502.5	380–869	0.5794
	100	887.0 ± 47.9	815.5	543–1187	748.4 ± 50.5	687.5	450–946	0.0583
	110	1001.9 ± 58.7	954.0	564–1297	879.0 ± 54.6	813.0	546–1099	0.0828
	120	805.2 ± 53.2	608.0	400–1220	880.7 ± 72.7	706.0	447–1146	0.7422
**Gap detection (ISI)**	1	**0.02 ± 0.03**	**−0.0008**	**−0.22 to 0.26**	**0.03 ± 0.03**	**0.06**	**−0.11 to 0.27**	0.5314
	2	0.09 ± 0.03	0.08	**−**0.13 to 0.32	**0.08 ± 0.02**	**0.11**	**−0.04 to 0.27**	0.4421
	5	0.24 ± 0.02	0.26	0.05–0.43	0.19 ± 0.02	0.23	0.03–0.38	0.1026
	10	0.44 ± 0.02	0.49	0.35–0.62	0.33 ± 0.02	0.37	0.21–0.49	0.0153*
	20	0.54 ± 0.02	0.59	0.41–0.69	0.38 ± 0.02	0.42	0.26–0.56	0.0011**
	40	0.23 ± 0.02	0.24	0.10–0.41	0.18 ± 0.03	0.27	0.03–0.42	0.3753
	80	0.10 ± 0.02	0.15	−0.04 to 0.35	0.14 ± 0.02	0.21	0.04–0.34	0.3789
	160	0.15 ± 0.02	0.17	0.01–0.38	0.13 ± 0.02	0.19	−0.01 to 0.36	0.6436
	240	0.15 ± 0.02	0.06	−0.03 to 0.35	0.13 ± 0.02	0.19	−0.02 to 0.34	0.7266
**90° Speaker swap (ISI)**	1	**−0.05 ± 0.05**	**−0.04**	**−0.24 to 0.21**	**−0.10 ± 0.04**	**−0.05**	**−0.35 to 0.15**	0.5659
	2	**0.04 ± 0.04**	**0.10**	**−0.16 to 0.27**	**−0.04 ± 0.05**	**0.04**	**−0.28 to 0.27**	0.3390
	5	**0.12 ± 0.05**	**0.19**	**−0.14 to 0.43**	**0.07 ± 0.05**	**0.12**	**−0.13 to 0.31**	0.5434
	10	0.27 ± 0.03	0.26	0.13–0.44	**0.11 ± 0.04**	**0.15**	**−0.02 to 0.33**	0.0823
	20	0.35 ± 0.03	0.39	0.18–0.56	**0.13 ± 0.05**	**0.19**	**−0.05 to 0.42**	0.0136*
	30	0.31 ± 0.04	0.37	0.12–0.54	**0.12 ± 0.05**	**0.16**	**−0.13 to 0.41**	0.0388*
	40	0.26 ± 0.03	0.28	0.10–0.46	**0.12 ± 0.05**	**0.18**	**−0.09 to 0.38**	0.1329
	80	0.32 ± 0.03	0.36	0.23–0.51	0.16 ± 0.04	0.24	−0.06 to 0.41	0.0809
	100	0.27 ± 0.04	0.32	0.09–0.48	0.20 ± 0.03	0.19	0.03–0.43	0.4565
	150	0.37 ± 0.03	0.42	0.19–0.56	0.23 ± 0.03	0.27	0.06–0.45	0.1400
	300	0.25 ± 0.03	0.25	0.11–0.43	**0.12 ± 0.04**	**0.15**	**−0.06 to 0.35**	0.1571
**Speaker swap (angle)**	7.5°	−**0.01 ± 0.02**	−**0.008**	**−0.21 to 0.22**	**−0.04 ± 0.03**	**0.06**	**−0.12 to 0.21**	0.4647
	15°	**0.04 ± 0.02**	**0.03**	**−0.14 to 0.26**	0.06 ± 0.02	0.10	**−**0.07 to 0.29	0.6043
	30°	**0.005 ± 0.03**	**0.04**	**−0.18 to 0.23**	**0.02 ± 0.02**	**0.08**	**−0.12 to 0.26**	0.6542
	45°	0.10 ± 0.03	0.17	−0.14 to 0.36	0.13 ± 0.02	0.17	0.010–0.37	0.3635
	90°	0.22 ± 0.02	0.24	0.08–0.40	0.12 ± 0.02	0.18	−0.01 to 0.37	0.0228*
**Threshold SRM (dB attenuation)**	9	0.40 ± 0.02	0.42	0.25–0.56	0.27 ± 0.03	0.30	0.19–0.44	0.0309*
	12	0.48 ± 0.02	0.55	0.33–0.65	0.31 ± 0.03	0.34	0.23–0.47	0.0067**
	15	0.49 ± 0.02	0.56	0.37–0.65	0.34 ± 0.02	0.36	0.23–0.51	0.0153*
	18	0.32 ± 0.03	0.32	0.21–0.47	0.29 ± 0.02	0.32	0.18–0.46	0.6152
	21	0.18 ± 0.03	0.21	0.07–0.38	0.17 ± 0.04	0.27	−0.03 to 0.39	0.7820
	24	**0.05 ± 0.03**	**0.10**	−**0.09 to 0.25**	0.09 ± 0.03	0.08	−0.11 to 0.33	0.5292
	27	**−0.04 ± 0.04**	**0.04**	**−0.23 to 0.19**	**0.05 ± 0.04**	**0.12**	**−0.11 to 0.32**	**0.1145**
**SRM 15 dB attenuation** **(angle)**	7.5°	0.42 ± 0.03	0.46	0.23–0.61	0.34 ± 0.03	0.40	0.23–0.48	0.2315
	15°	0.38 ± 0.03	0.35	0.20–0.61	0.30 ± 0.03	0.33	0.17–0.49	0.2133
	22.5°	0.36 ± 0.03	0.37	0.22–0.58	0.33 ± 0.03	0.40	0.17–0.49	0.6620
	30°	0.34 ± 0.03	0.40	0.19–0.57	0.31 ± 0.02	0.35	0.21–0.47	0.6156
	45°	0.41 ± 0.03	0.45	0.23–0.61	0.31 ± 0.03	0.35	0.15–0.47	0.1220
	90°	0.42 ± 0.03	0.49	0.27–0.64	0.21 ± 0.04	0.31	0.04–0.43	0.0030**
**SRM 24 dB attenuation** **(angle)**	7.5°	**−0.02 ± 0.04**	**−0.002**	**−0.24 to 0.19**	**0.07 ± 0.04**	**0.11**	**−0.09 to 0.35**	**0.1804**
	15°	**−0.05 ± 0.03**	**0.07**	**−0.16 to 0.28**	**0.06 ± 0.04**	**0.13**	**−0.07 to 0.28**	**0.8865**
	22.5°	**−0.006 ± 0.03**	**0.009**	**−0.19 to 0.18**	**−0.02 ± 0.05**	**0.11**	**−0.16 to 0.30**	**0.8580**
	30°	**0.02 ± 0.04**	**0.07**	**−0.20 to 0.28**	**0.04 ± 0.04**	**0.08**	**−0.11 to 0.31**	**0.7420**
	45°	**0.03 ± 0.04**	**0.13**	**−0.20 to 0.28**	**0.04 ± 0.04**	**0.09**	**−0.11 to 0.32**	**0.8239**
	90°	0.18 ± 0.03	0.24	0.06–0.38	0.18 ± 0.03	0.23	−0.03 to 0.43	0.9639

Values in bold indicate a PPI not significantly different from zero.

**Table 3:** Summary statistics for latency (ms)

		*B6* mice			*Fmr1* mice			
Experiment		Mean ± SEM	Median	IQR Q1–Q3	Mean ± SEM	Median	IQR Q1–Q3	*p* Value
**Threshold (dB SPL)**	60	47.2 ± 5.87	47.0	21.5–75.5	45.2 ± 7.6	47.0	18.0–63.0	0.8331
	70	41.9 ± 5.18	35.0	22.0–73.0	34.1 ± 6.3	26.0	15.3–41.5	0.2136
	80	36.6 ± 2.88	32.0	20.0–48.5	35.4 ± 3.5	32.0	18.3–43.5	0.7538
	90	25.4 ± 1.47	24.0	19.3–32.8	26.0 ± 1.9	24.0	18.0–33.0	0.8117
	100	25.1 ± 1.41	22.0	20.0–30.3	26.6 ± 2.0	24.5	21.0–32.0	0.6117
	110	25.0 ± 1.27	22.0	20.0–27.5	26.5 ± 1.7	24.0	20.0–31.0	0.5933
	120	26.9 ± 1.89	22.0	17.0–33.0	27.8 ± 2.4	23.0	18.0–34.5	0.7275
**Gap detection (ISI)**	1	26.6 ± 1.34	23.0	20.0–27.0	29.5 ± 1.48	26.0	19.8–33.0	0.0260*
	2	26.6 ± 1.38	23.0	17.0–29.0	30.9 ± 1.43	28.0	20.0–35.0	0.0057**
	5	28.9 ± 1.30	25.0	21.8–32.0	29.9 ± 1.35	27.0	18.0–36.0	0.1541
	10	34.2 ± 1.47	31.0	23.0–42.0	34.3 ± 1.74	28.5	20.0–45.0	0.3521
	20	35.7 ± 1.84	31.0	20.0–48.0	36.1 ± 1.88	30.0	20.0–51.5	0.3142
	40	28.2 ± 1.33	24.0	21.0–31.0	33.7 ± 1.61	29.0	20.0–43.5	0.0006***
	80	22.5 ± 0.53	23.0	21.0–24.3	30.4 ± 1.58	28.0	22.0–34.3	0.0002***
	160	21.7 ± 0.74	23.0	20.3–26.0	28.4 ± 1.51	28.0	20.8–33.0	0.0012**
	240	21.3 ± 0.84	22.5	19.0–25.0	32.0 ± 1.76	28.0	22.0–34.3	<0.0001***
**90° Speaker swap (ISI)**	1	23.2 ± 1.52	22.0	21.0–24.0	26.1 ± 1.68	25.0	22.0–32.0	0.2625
	2	21.6 ± 0.70	22.0	20.0–24.0	28.8 ± 2.15	26.0	21.0–33.0	0.0098
	5	22.3 ± 1.17	22.0	20.0–24.3	29.5 ± 2.06	25.5	22.0–36.8	0.0107*
	10	23.5 ± 1.46	24.0	20.0–29.0	30.9 ± 2.74	29.0	16.8–39.3	0.0100*
	20	23.0 ± 1.23	24.0	20.8–26.0	32.7 ± 2.78	29.0	23.0–39.0	0.0008***
	30	24.2 ± 1.06	24.0	21.0–30.3	34.5 ± 2.92	28.0	20.3–47.0	0.0005***
	40	24.6 ± 1.70	24.0	22.0–31.5	32.3 ± 2.40	30.0	22.0–40.0	0.0062**
	80	23.8 ± 1.23	24.0	20.8–30.3	33.2 ± 2.58	30.0	22.0–39.0	0.0013**
	100	22.3 ± 1.38	23.0	18.5–28.3	30.7 ± 2.87	28.0	14.0–41.0	0.0044**
	150	26.7 ± 1.68	26.0	22.0–31.5	32.2 ± 2.36	31.0	25.5–39.5	0.0489*
	300	23.4 ± 1.29	24.0	21.0–28.0	32.1 ± 2.25	29.0	29.0–23.0	0.0020**
**Speaker swap (angle)**	7.5°	21.5 ± 0.70	22.0	21.0–23.8	30.1 ± 1.30	27.0	22.0–34.0	<0.0001***
	15°	21.8 ± 0.79	21.0	20.0–23.8	29.4 ± 1.44	25.0	20.0–33.0	<0.0001***
	30°	22.0 ± 0.76	22.0	20.0–24.0	28.0 ± 1.22	25.0	21.0–33.0	<0.0001***
	45°	21.5 ± 0.69	22.0	20.0–24.0	31.1 ± 1.30	28.0	22.0–35.0	<0.0001***
	90°	24.9 ± 0.95	23.0	21.0–27.0	31.7 ± 1.56	27.0	20.8–35.3	<0.0001***
**Threshold SRM (dB attenuation)**	9	18.0 ± 2.42	10.0	5.0–22.5	36.1 ± 3.32	37.0	14.0–52.0	<0.0001***
	12	23.9 ± 2.78	15.0	10.0–31.0	34.0 ± 3.40	29.0	13.0–48.0	0.0231
	15	35.6 ± 2.50	31.0	19.5–53.5	36.6 ± 3.98	29.0	15.0–57.0	0.8374
	18	26.0 ± 1.87	24.0	21.0–29.8	35.8 ± 2.91	33.0	19.0–49.0	0.0224*
	21	25.4 ± 1.49	24.0	21.0–28.5	32.2 ± 2.92	28.5	17.5–42.0	0.0994
	24	23.4 ± 1.30	22.0	21.0–28.0	35.3 ± 2.44	31.0	24.0–45.0	0.0042**
	27	24.7 ± 1.67	22.0	21.0–25.0	30.3 ± 2.43	25.0	21.0–35.0	0.1750
**SRM 15 dB attenuation** **(angle)**	7.5°	25.4 ± 1.82	23.5	13.3–32.0	38.3 ± 3.49	32.0	18.0–57.5	0.0008***
	15°	27.8 ± 2.13	24.0	16.5–33.0	33.0 ± 3.06	31.0	12.5–43.5	0.1969
	22.5°	24.8 ± 1.97	23.0	14.0–30.0	29.6 ± 2.66	25.5	14.0–44.0	0.2396
	30°	23.5 ± 1.88	23.0	13.0–31.0	32.0 ± 3.45	26.0	11.0–48.0	0.0288*
	45°	25.5 ± 2.02	23.0	14.0–30.8	31.8 ± 3.29	24.0	14.0–41.0	0.1131
	90°	22.5 ± 2.52	13.0	8.5–29.5	29.0 ± 2.97	18.0	11.0–47.0	0.0803
**SRM 24 dB attenuation** **(angle)**	7.5°	23.6 ± 1.71	22.0	20.0–24.0	29.8 ± 2.33	25.0	22.0–34.0	0.0074**
	15°	25.3 ± 1.61	22.0	21.0–27.0	25.7 ± 1.98	23.5	14.0–32.0	0.5322
	22.5°	23.6 ± 1.51	22.0	21.0–24.0	32.0 ± 2.06	28.0	22.5–33.5	0.0004***
	30°	22.4 ± 1.46	22.0	21.0–24.0	30.1 ± 1.96	28.0	21.0–38.0	0.0014**
	45°	25.1 ± 1.62	22.0	20.0–27.0	30.8 ± 1.94	27.0	23.0–36.0	0.0098**
	90°	26.0 ± 1.61	24.0	24.0–29.0	26.3 ± 2.45	23.5	11.3–32.0	0.5437

## Results

### ABR audiogram

**Figure 1.**
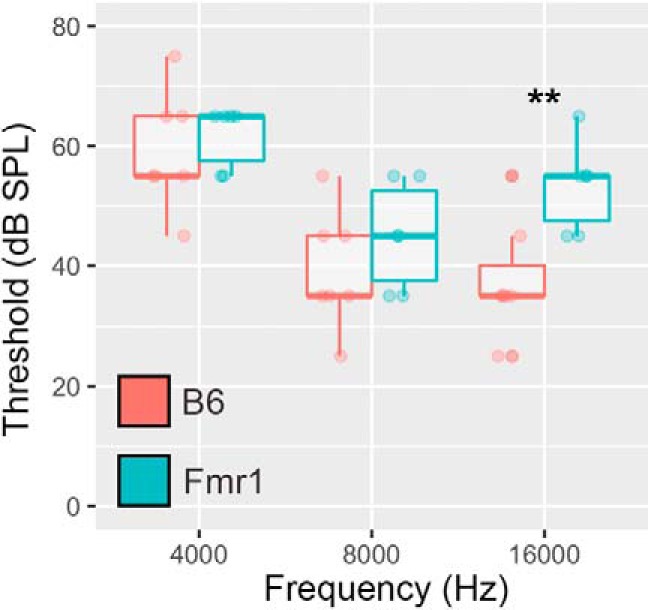
Increased auditory ABR threshold in *Fmr1* mice at 16 kHz compared with B6 mice. Tonal ABR measurements were made on mice at the following three frequencies: 4000, 8000, and 16,000 Hz. There were no differences in ABR auditory thresholds at 4000 and 8000 Hz between *Fmr1* and B6 mice. However, at 16 kHz *Fmr1* mice had increased thresholds compared with B6 mice. **p < 0.01.

The hearing range for *Fmr1* and B6 mice was determined through presenting tones of varying frequency (4, 8, 16 kHz) and recording the threshold of the ABR. These frequencies span the bandwidth of the noise stimuli used for behavior. There was a main effect of genotype (*p* = 0.043) indicating that the ABR thresholds were different between *Fmr1* and B6. *Post hoc* analysis showed that the two genotypes were no different for 4 and 8 kHz but that *Fmr1* mice had increased ABR thresholds at 16 kHz compared with wild-type mice (Fig. 1, Table 1).

### Experiment 1: startle threshold

**Figure 2.**
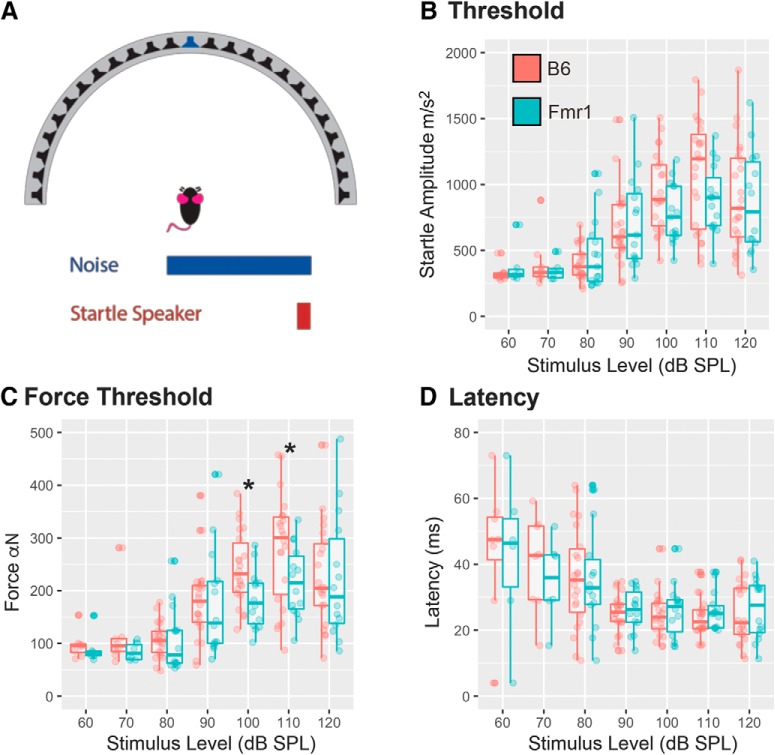
No difference in startle amplitude or latency between B6 and *Fmr1* mice decreased force in *Fmr1* mice. ***A***, Illustration of the experimental setup. Animals were placed in the center of the speaker array in the presence of 70 dB SPL broadband noise playing from the central speaker at 0° (blue speaker). Startle-eliciting stimuli were presented by an additional speaker placed directly over the head of the mouse (data not shown). ***B***, The animal’s startle amplitude increased with stimulus level (dB SPL) for both B6 mice (red) and *Fmr1* mice (teal). A total of 22 B6 mice and 14 *Fmr1* mice were tested in this task, with a subset (6 B6 mice; 8 *Fmr1* mice) tested <80 dB SPL. ***C***, The force of response to the startle stimuli was smaller for *Fmr1* mice compared with B6 mice. ***D***, Latency to respond (in ms) to the startle stimulus decreased with increasing stimulus intensity (dB SPL) for both B6 and *Fmr1* mice. *p < 0.05.

Animals were initially tested to determine their response threshold to acoustic startle stimuli. This was done both to characterize the responses of *Fmr1* mice and to ensure that all animals had a robust startle response with increasing intensity of sound. Startle amplitude is reported in units of arbitrary volts (output of the accelerometer) that are an uncalibrated measure proportional to acceleration. Startle sounds were 20 ms in duration and varied in intensity between 60 and 120 dB SPL presented randomly in 10 dB SPL steps, in the presence of continuous 70 dB SPL broadband noise (presented from the speaker directly in front of the animal; Fig. 2*A*). All animals were tested with intensities ranging from 80 to 120 dB SPL. Two additional levels (60 and 70 dB SPL) were tested in a subset of animals (6 B6 mice, 8 *Fmr1* mice) to ensure that animals are not startled at lower-intensity sounds. There was no difference between genotypes for either startle amplitude (Fig. 2*B*; *p* = 0.4074) or latency (Fig. 2*D*; *p* = 0.8331). Startle responses increased and latency decreased with increasing stimulus level in both genotypes, indicating that animals had no trouble detecting the startle stimulus and had a robust startle response. The magnitude of the startle responses for both B6 and *Fmr1* animals plateaued (threshold) at ∼100 dB SPL, and therefore the startle stimulus was set at 10 dB above this threshold (110 dB SPL) for the remainder of the experiments.

When individual animal weights were used to calculate the force of each startle response (in arbitrary units proportional to newtons), significant differences were observed between genotypes at 100 dB SPL (*p* = 0.0321) and 110 dB SPL (*p* = 0.0224) with *Fmr1* mice showing reduced startle force compared with wild-type mice (Fig. 2*C*). This could be due to a reduced muscle tone in *Fmr1* animals or some other factor, neither of which are explored further in this study. To account for differences in animal weight and reduced startle force, responses are normalized to the baseline startle amplitude when calculating PPI (see Materials and Methods).

### Experiment 2: gap detection

**Figure 3.**
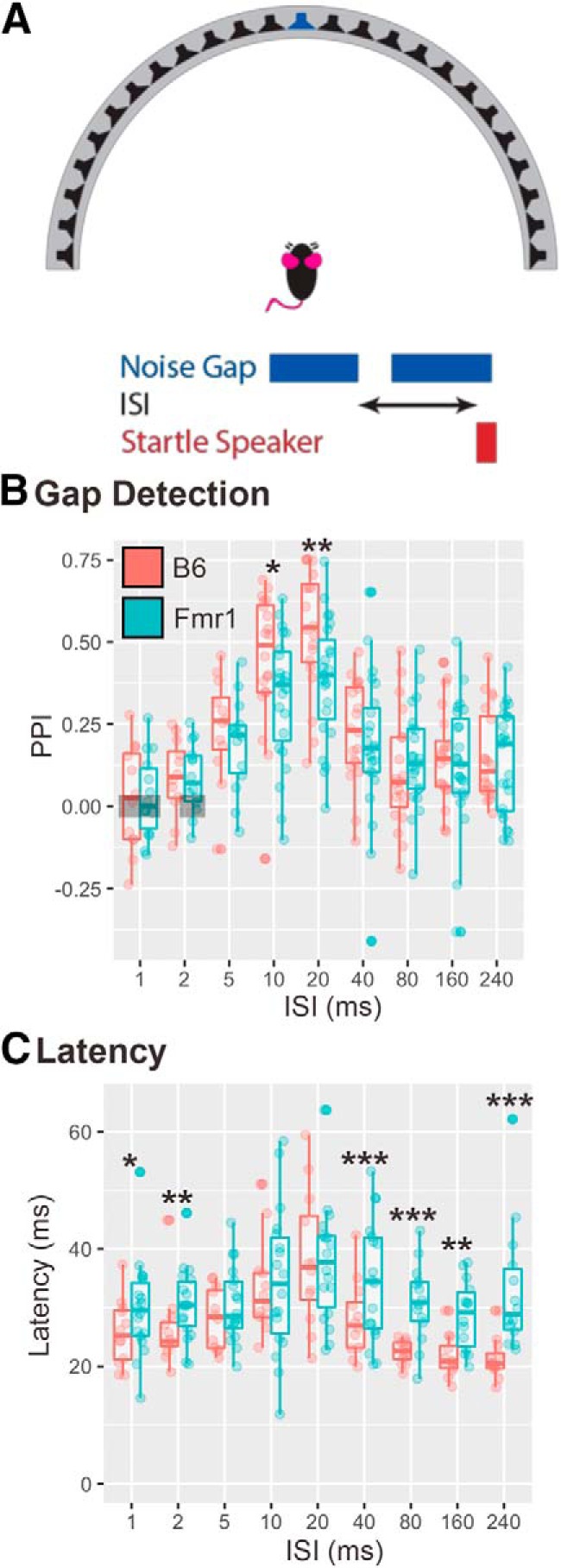
*Fmr1* mice have decreased PPI and increased latency to startle during a gap detection task. ***A***, Forty-two mice (20 B6 mice; 22 *Fmr1* mice) were tested in a gap detection task, where a 20 ms quiet gap was introduced into 70 dB SPL broadband noise (blue speaker at the center of the array), which was presented at varying ISIs before the startle-eliciting stimulus presentation. ***B***, *Fmr1* mice (teal) showed less PPI at 10 and 20 ms ISI compared with B6 mice (red). ***C***, *Fmr1* mice startle responses latencies were longer to 1, 2, 5, 40, 80, 160, and 240 ms ISIs compared with wild-type mice. Boxes show conditions where PPI is not different from zero for both genotypes. *p < 0.05, **p < 0.01, ***p < 0.001.

Recent data have shown that impairments in gap detection may be caused by underlying changes to E/I balance in the inferior colliculus (IC; Sturm et al., 2017), suggesting that gap detection may be used to probe the E/I balance in the auditory system. This E/I balance is known to be altered in FXS (Rotschafer et al., 2015; Garcia-Pino et al., 2017; McCullagh et al., 2017). We tested 42 animals (20 B6 mice, 22 *Fmr1* mice) in a gap detection paradigm with a 20 ms quiet gap in broadband noise (prepulse) followed by a startle-eliciting stimulus at varying ISI times (1–240 ms) between the prepulse and startle-eliciting stimuli (Fig. 3*A*,*B*). A subset of animals was only tested at 10–240 ms ISIs (8 B6 mice, 8 *Fmr1*mice). There was no main effect of genotype (*p* = 0.2011) at all durations of ISI; however, there were significant differences between B6 and *Fmr1* animals at 10 ms (*p* < 0.05) and 20 ms (*p* < 0.01) ISIs (Fig. 3*B*). In addition, *Fmr1* mice were slower to startle at all ISIs (as indicated by increased startle latency; main effect of genotype, *p* = 0.00000255), except at 10 and 20 ms, compared with B6 mice (Fig. 3*C*). Both genotypes did not show PPI significantly different from zero for ISI of <1 ms (*p* = 0.45, B6 mice; *p* = 0.91, *Fmr1* mice), and additionally at 2 ms (*p* = 0.14) for *Fmr1* mice, suggesting a lack of detection of the prepulse with these short ISIs. These data suggest that not only do *Fmr1* mice have decreased PPI compared with wild-type mice at optimal ISIs but are also consistently slow to startle under most conditions, where in contrast, B6 mice show modulations to latency based on ISI.

### Experiment 3a: varying ISI with 90° speaker swap

**Figure 4.**
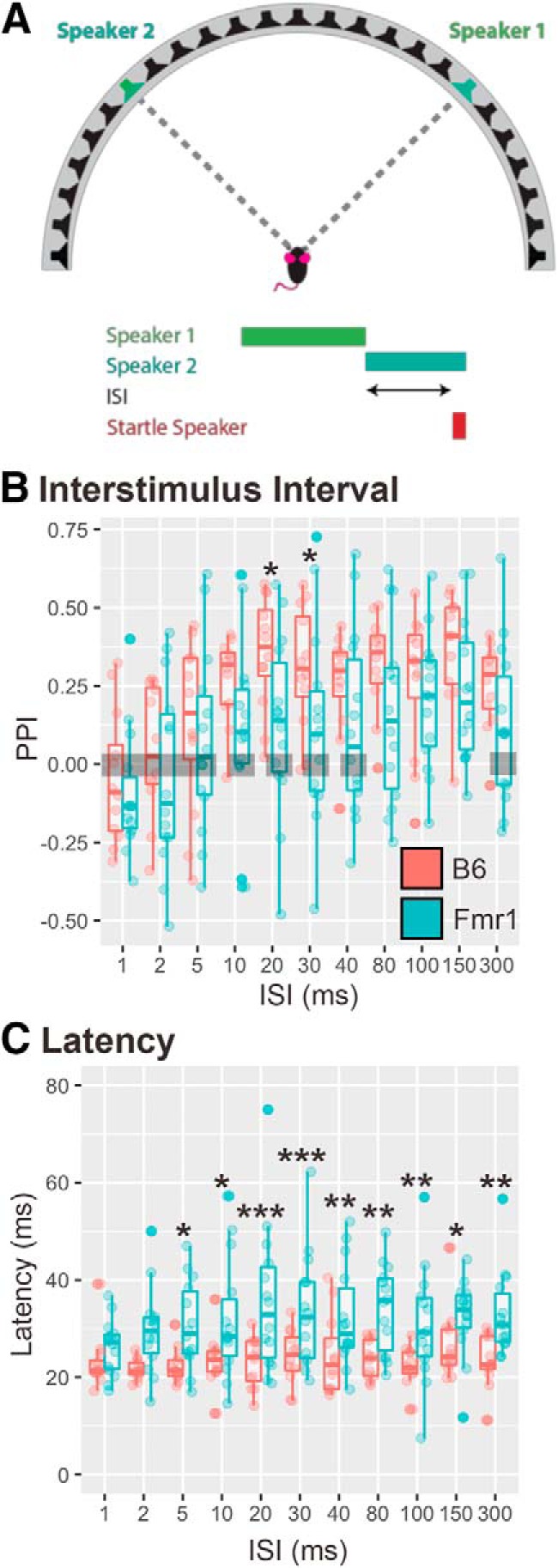
*Fmr1* mice have decreased PPI and increased startle latency in response to a 90*°* speaker swap compared with B6. ***A***, Twenty-six mice (12 B6 mice; 14 *Fmr1* mice) were tested in a 90*°* angle speaker swap task with varying ISI. ***B***, *Fmr1* mice (teal) have lower PPI at 20 and 30 ms ISIs than B6 mice (red). *Fmr1* mice had longer latencies to startle at all ISIs except 1 and 2 ms compared with B6 mice. Boxes show conditions where PPI is not different from zero for both genotypes. *p < 0.05, **p < 0.01, ***p < 0.001.

The gap detection test suggests that *Fmr1* mice demonstrated deficits in temporal auditory processing, next we wanted to determine whether *Fmr1* mice also have deficits in spatial auditory processing. The first step to determining whether *Fmr1* mice have spatial hearing deficits is to establish the optimal ISI for the detection of a spatial speaker swap. In this task, the prepulse was a speaker swap of broadband noise from one speaker 45*°* to the right of the animal to the symmetrical speaker 45*°* to the left of the animal (90*°* total angle), with varying ISIs between the prepulse and startle-eliciting stimuli (Fig. 4*A*). There was no main effect of ISI on genotype (*p* = 0.1068). However, *Fmr1* mice had reduced PPI of their startle compared with B6 mice at 20 and 30 ms (*p* < 0.05) ISI after the 90*°* speaker swap (12 B6, 14 *Fmr1* mice; Fig. 4*B*). In addition, *Fmr1* mice showed an increased latency to startle compared with B6 at all ISIs except for 1 and 2 ms (Fig. 4*C*; main effect, *p* = 0.0000007913). These data suggest that at ISIs that elicited some of the highest PPI values for B6 animals (also similar ISIs that showed a deficit in the gap detection test), *Fmr1* mice showed reduced PPI compared with wild-type mice. Neither genotype demonstrated PPI significantly different from zero for ISIs ≤5 ms (1, 2, or 5 ms ISIs), suggesting a lack of detection at these ISIs (B6: 1 ms, *p* = 0.50; 2 ms, *p* = 0.51; 5 ms, *p* = 0.07; *Fmr1*: 1 ms, *p* = 0.13; 2 ms, *p* = 0.50; 5 ms, *p* = 0.27). *Fmr1* animals did not have PPI significantly different from zero for ISIs of 10 ms (*p* = 0.079), 20 ms (*p* = 0.05), 30 ms (*p* = 0.068), 40 ms (*p* = 0.06), and 300 ms (*p* = 0.059), also suggesting potential impaired detection at these ISIs. In addition, *Fmr1* mice showed increased latencies to startle at ISIs that elicited a PPI >0, suggesting that the addition of a detectable prepulse actually slowed responses compared with B6 mice. Based on the results of this task and the gap detection, it was determined that the optimal ISI for spatial tasks for B6 mice is 20 ms, which is consistent with other studies (Allen and Ison, 2010).

### Experiment 3b: minimum audible angle detection with a fixed ISI

**Figure 5.**
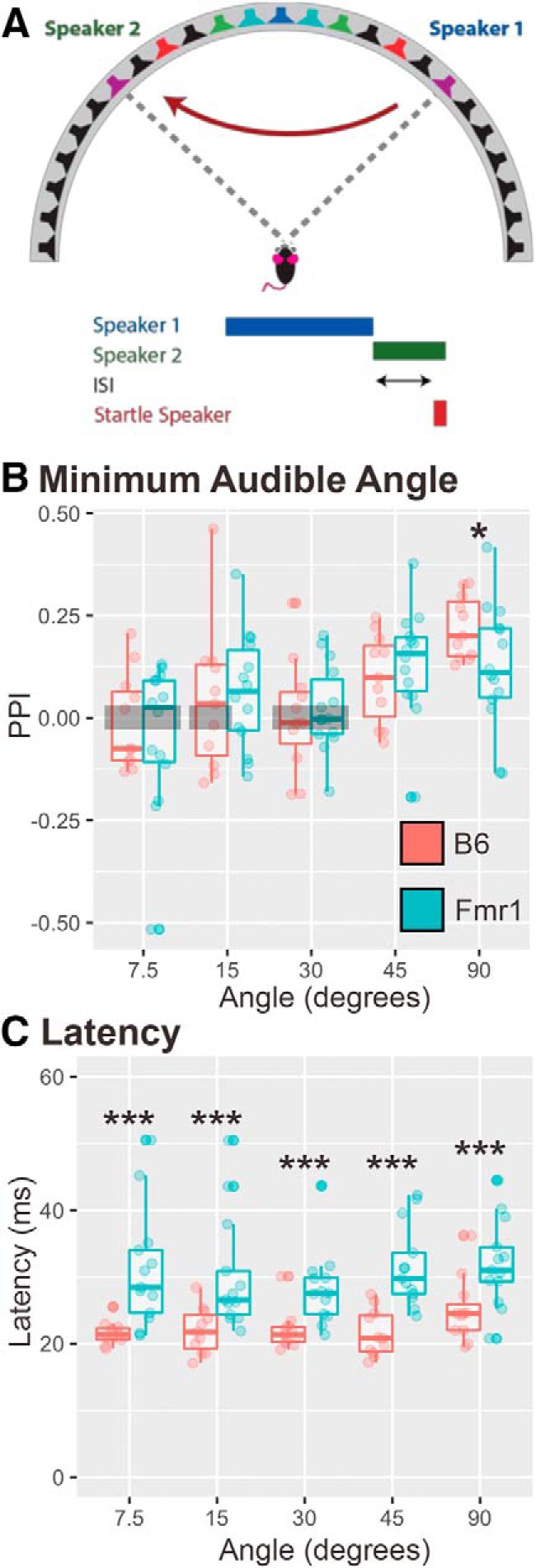
*Fmr1* mice had a reduction in PPI compared with B6 mice at 90*°* speaker swap and longer latencies to startle in all conditions. ***A***, Twenty-five mice (11 B6 mice; 14 *Fmr1* mice) were tested with varying angles of a speaker swap, both left and right directions across the midline, and constant 20 ms ISI (example leftward swap is shown). ***B***, *Fmr1* mice (teal) show less PPI at 90*°* than B6 mice (red). ***C***, *Fmr1* mice had longer latencies to startle at all angle conditions compared with B6 mice. Boxes show conditions where PPI is not different from zero for both genotypes. *p < 0.05, ***p < 0.001.

To determine whether *Fmr1* mice have impairments in spatial acuity, we measured and compared minimum audible angle detection for *Fmr1* and wild-type mice. The minimum audible angle is defined as the smallest change in speaker source location that the animals could just detect via the PPI metric (PPI significantly different from zero). In this task, the angle of the speaker swap across the midline was varied as the prepulse to the startle, with a constant ISI of 20 ms as established by the previous experiments. Twenty-five mice (11 B6 mice; 14 *Fmr1* mice) were tested with angle swaps (to the left and right of the animal) of 7.5°, 15°, 30°, 45°, and 90° across the midline (i.e., with the animal oriented toward 0*°*; Fig. 5*A*). Data were comparable for left to right and right to left directional swaps; therefore, the data were pooled for both directions (Fig. 5*B*,*C*). There was no main effect of genotype in this task (*p* = 0.7255). *Fmr1* mice showed less PPI than B6 mice only at the 90*°* angle speaker swap, suggesting that minimum audible detection was comparable in the two groups (Fig. 5*B*). Neither genotype showed PPI significantly different from zero for angles of ≤30*°* (except for at 15°, where *Fmr1* mice show PPI > 0, *p* = 0.026), suggesting that at these angles the animals could not detect the speaker swap, and that the minimum audible angles of the animal were less than ∼45° (B6 mice: 7.5°, *p* = 0.75; 15°, *p* = 0.19; 30°, *p* = 0.89; *Fmr1* mice: 7.5°, *p* = 0.15; 30°, *p* = 0.41). These data suggest that both genotypes have poor angular discrimination abilities as assessed in this task; however, *Fmr1* mice had longer latencies to startle at all angles compared with B6 mice (Fig. 4*C*; main effect, *p* < 0.0001), which is consistent with results in the previous varying ISI experiment.

### Experiment 4a: auditory spatial release from masking threshold signal detection in noise

**Figure 6.**
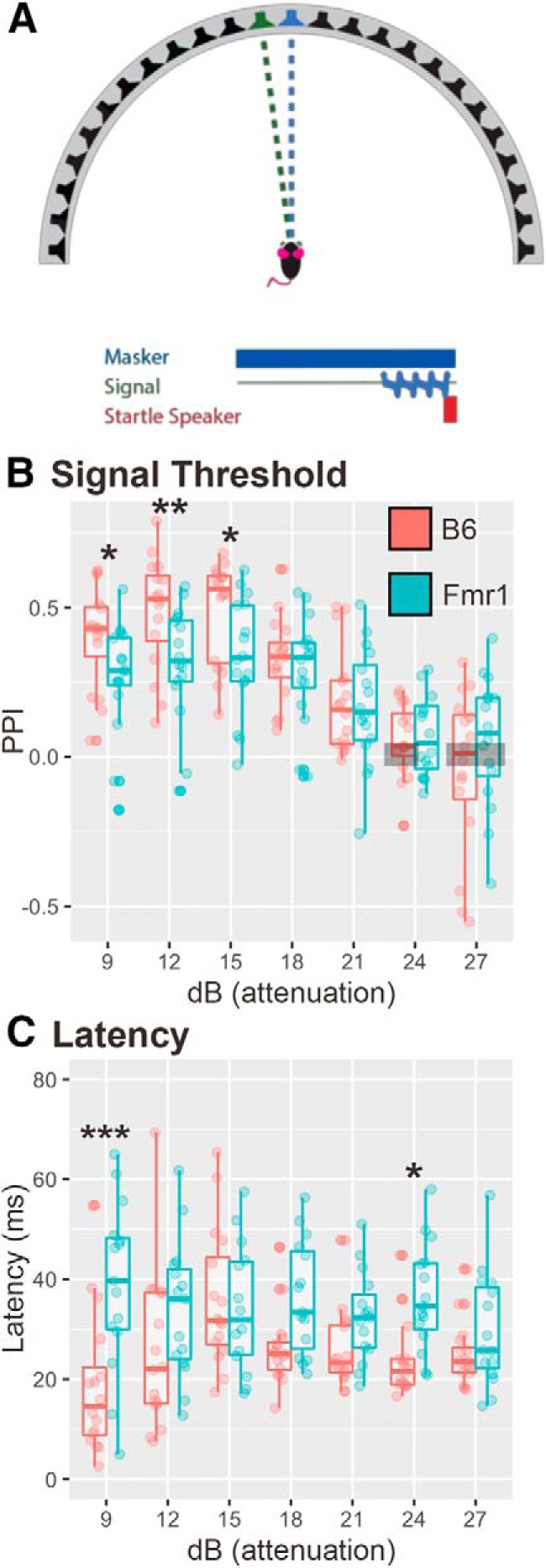
*Fmr1* mice showed less PPI of their startle, and longer latencies in some conditions compared with B6 mice. ***A***, A signal noise was played at varying sound levels from a loudspeaker offset by 7.5° from a 70 dB SPL masker noise at 0°. ***B***, *Fmr1* mice (teal) had reduced PPI of their startle at 9, 12, and 15 dBs attenuation compared with B6 mice (red). ***C***, *Fmr1* mice also had increased latency to startle at 9 and 24 dB attenuation compared with B6 mice. Boxes show conditions where PPI is not different from zero for both genotypes. *p < 0.05, **p < 0.01, ***p < 0.001.

Listening to sounds in a complex auditory environment with competing sound sources (spatial release from masking) more naturally replicates real-world listening environments, in which both people and mice with FXS experience difficulties. We used a PPI-based task to replicate this experience, and to determine whether *Fmr1* mice have impairments in SRM. First, we determined the signal attenuation required for animals to no longer be able to distinguish signals from the background. Signal detection thresholds were measured in 32 animals (16 B6 mice; 16 *Fmr1* mice) placed in the chamber with a 70 dB SPL masker sound presented from the 0° speaker, and a prepulse cue centered around 4 kHz was played at varying attenuated levels (from ∼83.5 dB SPL) from the adjacent speakers (7.5° to the left or right; Fig. 6*A*). A tone-based sound was chosen because it is audible to the animal while not eliciting a social or emotional response (e.g., vocalization sounds) that might elicit an unexpected behavioral response. In this task, there was no main effect of genotype (*p* = 0.2786). At the loudest levels (9–15 dB attenuation), the 4 kHz sound elicited a robust PPI in both genotypes, although *Fmr1* mice showed less PPI of their startle response compared with B6 mice (Fig. 6*B*). Both genotypes did not have PPI >0 at 27 dB attenuation (B6, *p* = 0.31; *Fmr1*, *p* = 0.22), and B6 animals also at 24 dB attenuation (*p* = 0.22), suggesting that mice did not detect the prepulse at these levels, and that their detection threshold was <21–24 dB attenuation. Similar to latencies in the gap detection task, B6 mice showed modulation of their latency based on condition, whereas *Fmr1* mice had consistent slower latencies across conditions (Fig. 6*C*; main effect genotype, *p* = 0.001189).

### Experiment 4b: spatial release from masking varying angle

**Figure 7.**
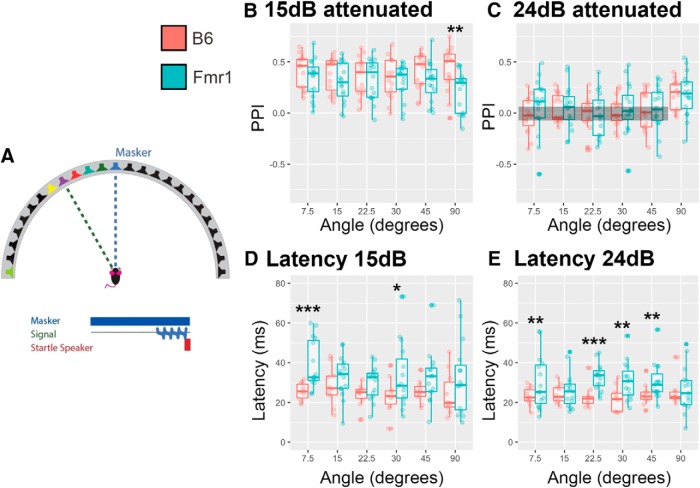
*Fmr1* mice had reduced PPI at 90° and 15 dB attenuation with increased latency to startle in several conditions compared with B6 mice. ***A***, The signal was varied at two levels of attenuation (15 and 24 dB) and varied at several angles from the 70 dB SPL masker at 0° to the left or right. ***B–E***, *Fmr1* mice (teal) had decreased PPI of their startle response at 90° from the masker only at 15 dB attenuation compared with B6 mice (red; ***B***, ***C***). *Fmr1* mice had increased latency to startle at 7.5° and 30° at 15 dB attenuation (***D***) and at 7.5°, 22.5°, 30°, 45°, and 90° at 24 dB attenuation (***E***) compared with B6 mice. Boxes show conditions where PPI is not different from zero for both genotypes. *p < 0.05, **p < 0.01, ***p < 0.001.

Next, to determine whether *Fmr1* mice had impairments in their spatial release from masking, the angular separation between masker and prepulse signal was varied. In this task, 31 animals (15 B6 mice; 16 *Fmr1* mice) were presented with the same 4 kHz signal (prepulse), at two attenuation levels (15 and 24 dB attenuation), at varying angles relative to a masker noise presented from the 0° speaker (Fig. 7*A*). There was no main effect of genotype at either 15 dB attenuation (*p* = 0.1267) or 24 dB attenuation (*p* = 0.6325). Consistent with the speaker swap task, *Fmr1* mice only showed a difference in PPI relative to B6 mice at the largest angle (90°), and only at the louder (15 dB attenuation) level (*p* < 0.05; Fig. 7*B*,*C*). Both *Fmr1* and B6 mice showed PPI >0 at all angles for the louder signal (15 dB attenuation, *p* > 0.05), suggesting that the signal was readily detected above the masker at all angles, and only showed PPI >0 for the largest angle (90°) for the quieter signal (24 dB attenuation; *p* < 0.05 at 90° and *p* > 0.05 for all other angles for both genotypes). Similar to the above experiments, *Fmr1* mice had longer latencies to startle compared with B6 mice at several angles (7.5° and 30° at 15 dB attenuation; and 7.5°, 22.5°, 30°, 45° at 24 dB attenuation; Fig. 7*D*,*E*; main effect genotype, *p* = 0.0001). These data do not indicate substantial deficits in SRM; however, the longer latencies seen here, and in other experiments, suggest altered timing of startle responses in *Fmr1* mice compared with wild-type mice.

### Habituation of the startle response

Previous studies have shown that *Fmr1* mice have impaired habituation (decreased startle response or PPI in later presentations of a replicate) during PPI tasks (Nielsen et al., 2002). Therefore, we used the replicate number per condition (e.g., ISI, dB) as the main effect variable per genotype. In contrast to previous studies, we found no habituation in either the B6 or *Fmr1* knock-out mice in any of the experiments tested (startle threshold, gap detection, speaker swaps, or spatial release from masking; Nielsen et al., 2002). This was indicated as no change in startle amplitude or PPI as a result of replicate, in particular the second replicate compared with the last replicate per condition block (*p* > 0.05 in all tests).

## Discussion

We characterize the binaural and spatial hearing ability of *Fmr1* mice using a reflexive PPI task. In addition, we measured the audiogram of mice using the ABR. Surprisingly, *Fmr1* mice appear to show spatial hearing ability comparable to wild-type mice, with only subtle differences noted in some prepulse conditions. *Fmr1* mice showed similar “detection” ability (PPI >0) of prepulses under most conditions compared with wild-type mice, except for varying the ISI with 90° swap. In contrast, *Fmr1* mice had increased latency to startle in all experiments except when determining the startle threshold. These data suggest that perhaps *Fmr1* mice do not have a severe spatial hearing deficit but do show impairments in the timing of responses. Last, we did not see any short-term habituation response within experiments.

### Increased high-frequency ABR thresholds

*Fmr1* mice had somewhat increased ABR thresholds at 16 kHz compared with B6 mice, indicating potential high-frequency hearing loss. Previous studies examining ABR in *Fmr1* mice saw no change in latency of the ABR waveforms and a reduction in wave I amplitude (Rotschafer et al., 2015; El-Hassar et al., 2019). One study saw an increase in ABR threshold (though across all frequencies) in *Fmr1* mice (Rotschafer et al., 2015), while the other saw no change in ABR threshold based on frequency (El-Hassar et al., 2019). However, both of these previous ABR studies were performed in the *Fmr1* FVB knock-out strain, which is one potential reason to explain the difference in results with our study. In addition, the mice used in our study were slightly older, with an average age of 125 d for both genotypes, though we did not see any obvious age-related hearing loss in either genotype (as indicated by reduced thresholds overall across frequencies). Last, these ABR experiments were performed independently of the PPI experiments described previously in this study; therefore, it is difficult to know whether the results found here are directly related to changes we see in PPI.

### No change in overall startle threshold

Our results indicate that there is no difference in the overall acoustic startle response between *Fmr1* mice and B6 mice. However, startle force, accounting for the weight of the mice, did show differences at 100 and 110 dB SPL, suggesting that *Fmr1* mice startle with less force. Accounting for the mass of an animal could help interpretation of the data across experimental methods and apparatuses (Grimsley et al., 2015). None of the previous studies account for the weight or force of the animal, and the results in *Fmr1* mice are equivocal, where some studies show an increase in the ASR (Nielsen et al., 2002; Arsenault et al., 2016), while others show a decrease in the ASR (Chen and Toth, 2001; Nielsen et al., 2002; Frankland et al., 2004; Spencer et al., 2006; Paylor et al., 2008; Baker et al., 2010; Thomas et al., 2012; Veeraragavan et al., 2012; Yun et al., 2006), and still others show no change in overall ASR (Ding et al., 2014), which is consistent with our study. The cause of these discrepancies is not clear; however, one possible explanation is the use of different mouse strains between previous studies (Bullock et al., 1997). Additional differences across reports include the experimental setup and the method of measuring the ASR.

Studies comparing patients with FXS and neurotypical human subjects did not find any differences in startle magnitude (Frankland et al., 2004; Hessl et al., 2009) consistent with our results. Last, studies have shown that the ASR is related directly to FMRP expression (Yun et al., 2006) and can be rescued with addition of the *Fmr1* gene (Paylor et al., 2008) suggesting that some aspects of the ASR are directly related to loss of FMRP.

### Fmr1 mice show decreased PPI during 10 and 20 ms gaps

Gap detection ability is thought to be directly related to E/I balance in the auditory system, particularly in the IC (Sturm et al., 2017). E/I imbalances have been found in *Fmr1* mice in the auditory brainstem, particularly the medial nucleus of the trapezoid body (Rotschafer et al., 2015; McCullagh et al., 2017) and lateral superior olive (Garcia-Pino et al., 2017). These areas also contribute to the PPI and ASR pathways as they convey sound location information to higher areas such as the IC (Koch, 1999). Our data show that *Fmr1* mice show lower PPI at short gap lengths (10 and 20 ms), suggesting that *Fmr1* mice have impairments in their inhibition of the startle response. However, gap detection is dependent on high-frequency hearing ability, and, as indicated by the ABR audiogram, *Fmr1* mice have higher ABR thresholds at 16 kHz compared with B6 mice (Fitzgibbons and Gordon‐Salant, 1987). High-frequency hearing difficulties could help explain some of the deficits that we see in the *Fmr1* knock-out mice. In addition, our study examined latency to startle, and, interestingly, *Fmr1* mice did not show the reduction in startle latency at gap ISIs >20 ms observed in wild-type mice.

### Fmr1 mice show decreased PPI to 90° speaker swap

This is the first study to measure the minimum audible angle detection of *Fmr1* mice. *Fmr1* mice showed less PPI at any ISI with a 90° speaker swap, indicating that they had overall lower-magnitude startle responses even at such a large angle than wild-type mice (only significantly different from wild-type mice at 20 and 30 ms). In addition, when we kept the ISI at 20 ms and varied the angle, *Fmr1* mice again showed lower PPI values at 90° speaker swaps compared with B6 mice. There was no difference in pinna morphology, which is one possible explanation for the differences seen in 90° speaker swaps. However, both genotypes showed low PPI values for other speaker angles, suggesting that these mice exhibit poor minimum audible angle ability. Other studies have shown higher PPI values for mice at smaller angle swaps than we report; however, differences in background strain (CBA/CaJ and CBA/129) and stimuli presented (wide-band noise vs high-pass noise, >4 kHz) may explain the differing results (Allen and Ison, 2010; Lauer et al., 2011). Moreover, the upper frequency of noise we used was ∼20 kHz. In addition, consistent with other experiments reported here, the *Fmr1* mice responded with longer latencies to startle at almost all angles compared with B6 mice. This indicates that not only do *Fmr1* mice have difficulties in inhibiting their startle response, but *Fmr1* mice may have longer processing speeds than B6 mice.

### Fmr1 mice show alterations to spatial release from masking

Spatial release from masking involves detecting a signal in a noisy background. We show that even at loud signals compared with background, *Fmr1* mice show less PPI of their startle response than B6 mice. In addition, when varying the location of the signal, at the louder sound, mice again had deficits at 90°, but this time off to the side of the animal. Last, similar to the speaker swap experiments, *Fmr1* animals also had longer latencies under most conditions to respond to the startle speaker compared with B6 animals, suggesting again not only impairments in detection, but also reaction ability/time.

Other studies have examined PPI while varying the intensity of a prepulse signal above an ambient noise level. While not exactly the same as the SRM task discussed here, in contrast to our results, most studies found that *Fmr1* mice had increased PPI compared with wild-type mice (Chen and Toth, 2001; Nielsen et al., 2002; Frankland et al., 2004; Paylor et al., 2008; Baker et al., 2010; Veeraragavan et al., 2012; but see Spencer et al., 2006; Thomas et al., 2012). These discrepancies could be due to the prepulse eliciting a startle response in these other studies, which would cause increased PPI during the actual startle, and in particular since these studies did not explore latency to startle, the prepulse startle response could be delayed coinciding with the startle-eliciting speaker. Last, most of the other studies do not discuss where the signal is coming from, which could impact the inhibition of the startle response in these animals and is likely different from our experiments. Interestingly, our results are consistent with data from patients with FXS who show reduced PPI under similar conditions (Frankland et al., 2004; Hessl et al., 2009). Consistency with human data implies that our assay may be a better measure of PPI that could apply to drug rescue experiments and be more applicable to the human FXS condition.

### Mice in these experiments did not habituate

Often animals habituate to the startle stimulus, meaning that as the animal continues to be exposed to a loud sound stimulus, they will no longer startle as robustly as at the earlier presentations of the stimulus. Habituation can also limit the length of experiments since animals may not respond as robustly after several hours of testing. Interestingly, we did not see any habituation to the startle in either *Fmr1* or B6 animals, as seen by a change in PPI or startle amplitude between early and later presentations of the same stimulus for any of the tasks presented. Other studies have examined habituation and shown that *Fmr1* mice do not habituate, though their results were not consistent between an F1 cross of genotypes and *Fmr1* mice on a B6 background, suggesting that their results might be a result of background genotype (Nielsen et al., 2002). Mice typically show less habituation than other animals, responding robustly and consistently to many stimulus presentations, and habituation can be extinguished with a few minutes rest between experiments (Valsamis and Schmid, 2011). Our results suggest also that mice can tolerate several hours (we kept total testing time under 3 h) of testing without a concern for habituation to the startle response, particularly in the B6 background strain tested here.

### Latency versus startle

In contrast to PPI and ASR responses that differed between *Fmr1* and B6 mice, where we saw specific impairments under certain conditions, there was an overall trend for *Fmr1* mice to have increased latency to startle under a variety of conditions. This could be due to impairments in a different circuit that causes the response to the startle (i.e., when compared with how much to startle in *Fmr1* mice). There has been one study that examined the latency to react in patients with FXS after an acoustic startle, and they saw no differences between neurotypical control subjects and FXS patients (Roberts et al., 2013). This study, however, did not use PPI as a measure or look at EMG responses to the acoustic startle. None of the other studies examining PPI and ASR in *Fmr1* mice or humans examined the latency to respond, making it difficult to know whether our results are comparable. More in-depth gap detection experiments and a more thorough ABR characterization could shed light on the latency changes seen here in this study, though Rotschafer et al. (2015) did not see any changes to latency of the ABR (however, in a different background strain: FVB). In addition, the circuit underlying latency to respond to ASR or PPI is not well understood and is an interesting area for further exploration.

### Conclusions

Several recent studies described anatomic alterations in the sound localization pathway of *Fmr1* mice, which lead to altered physiologic properties (Brown et al., 2010; Strumbos et al., 2010; Rotschafer et al., 2015; Garcia-Pino et al., 2017; McCullagh et al., 2017; Rotschafer and Cramer, 2017; Curry et al., 2018; El-Hassar et al., 2019; Lu, 2019). These alterations include differences in synaptic strength and connectivity; differences in postsynaptic ion channels, postsynaptic input resistance, and altered firing properties; alterations in action potential shape; and differences in macroscopic physiologic properties such as ABRs. In a circuit in which amplitude, kinetics, and timing of excitation and inhibition are balanced very precisely to perform sound localization, these alterations should have dramatic effects on the localization ability of the animal. Surprisingly, the observed effects were smaller than expected and do not support the view of a degraded localization circuit “across the board.” However, our results are consistent with observations from human FXS patients, suggesting that the *Fmr1* mouse model can recapitulate the human FXS condition well, at least as far as the sound localization circuit is concerned.

## Synthesis

Reviewing Editor: Darcy Kelley, Columbia University Decisions are customarily a result of the Reviewing Editor and the peer reviewers coming together and discussing their recommendations until a consensus is reached. When revisions are invited, a fact-based synthesis statement explaining their decision and outlining what is needed to prepare a revision will be listed below. The following reviewer(s) agreed to reveal their identity: Amanda Lauer, Christine Portfors.

Effectively revised
